# Phytochemical Analysis and Biological Activities of *Wollemia nobilis* W.G.Jones, K.D.Hill & J.M.Allen Leaves Collected in the Botanical Garden of Rome

**DOI:** 10.3390/plants14081244

**Published:** 2025-04-19

**Authors:** Claudio Frezza, Daniela De Vita, Ottavia Giampaoli, Marzia Beccaccioli, Michela Verni, Federica Violetta Conti, Laura Fonti, Marco Franceschin, Fabio Sciubba, Claudio Scintu, Letizia Corsetti, Antonella Di Sotto, Carlo Giuseppe Rizzello, Massimo Reverberi, Fabio Attorre

**Affiliations:** 1 Dipartimento di Scienze della Vita, della Salute e delle Professioni Sanitarie, Università degli Studi Link Campus, Via del Casale di San Pio V, 44, 00165 Rome, Italy; 2 Dipartimento di Biologia Ambientale, Università di Roma “La Sapienza”, Piazzale Aldo Moro 5, 00185 Rome, Italy; daniela.devita@uniroma1.it (D.D.V.); ottavia.giampaoli@uniroma1.it (O.G.); marzia.beccaccioli@uniroma1.it (M.B.); michela.verni@uniroma1.it (M.V.); violetta.conti@uniroma1.it (F.V.C.); laurafonti49@gmail.com (L.F.); fabio.sciubba@uniroma1.it (F.S.); claudio.scintu@uniroma1.it (C.S.); carlogiuseppe.rizzello@uniroma1.it (C.G.R.); massimo.reverberi@uniroma1.it (M.R.); fabio.attorre@uniroma1.it (F.A.); 3 Dipartimento di Chimica, Università di Roma “La Sapienza”, Piazzale Aldo Moro 5, 00185 Rome, Italy; marco.franceschin@uniroma1.it; 4 NMR-Based Metabolomics Laboratory (NMLab), Università di Roma “La Sapienza”, Piazzale Aldo Moro 5, 00185 Rome, Italy; 5 Dipartimento di Fisiologia e Farmacologia “V. Erspamer”, Università di Roma “La Sapienza”, Piazzale Aldo Moro 5, 00185 Rome, Italy; letizia.corsetti@uniroma1.it (L.C.); antonella.disotto@uniroma1.it (A.D.S.)

**Keywords:** *Wollemia nobilis* W.G.Jones, K.D.Hill & J.M.Allen, leaves, Botanical Garden of Rome, phytochemical analysis, biological activities

## Abstract

In this work, a preliminary screening of the bioactivities of an ethanolic extract obtained from the leaves of *Wollemia nobilis* W.G.Jones, K.D.Hill & J.M.Allen was carried out to explore its potential pharmaceutical applications. In particular, the radical scavenging, chelating, reducing antiglycative, antimicrobial and antifungal activities as well as the inhibitory effects on the production of aflatoxin B1 in *Aspergillus flavus* Link were evaluated. The extract demonstrated promising biological activities, although generally with lower potency compared to the positive control. To identify the metabolites potentially responsible for these effects, the extract was subjected to phytochemical analysis evidencing the presence of eight known compounds. Among them, 15-agathic acid methyl ester (**1**) and ladanein (**5**) were reported for the first time in this species. Furthermore methyl-(*E*)-communate (**2**), 7,4′,7″,4‴-tetra-*O*-methyl-robustaflavone (**6**), agathisflavone (**7**) and quinic acid (**8**) were detected for the first time in the leaf tissue of *W. nobilis*. Their presence and the presence of isocupressic acid (**3**) and acetyl-isocupressic acid (**4**) in this species highlights the taxonomic correlations within the Araucariaceae family and suggests a possible contribution of these compounds in the bioactivities of the extract. However, further studies are required to confirm these contributions and to elucidate their mechanisms of action.

## 1. Introduction

*Wollemia nobilis* W.G.Jones, K.D.Hill & J.M.Allen is a coniferous tree belonging to the Araucariaceae family, native to Queensland (Australia), where it was recently rediscovered after being believed extinct for centuries. Nowadays, it has been introduced in several countries of the world to favor its worldwide repopulation, including the Botanical Garden of Rome, where seeds from the Australian trees have been planted.

In these last years, different and separated organs of this species have been studied by our group for their non-volatile phytochemical composition, evidencing different classes of natural compounds, especially diterpenoids, biflavonoids, flavonoids and simple phenolics [1,2,3,4,5,6], but no biological assay has been performed on them. Actually, no biological assay has ever been performed on the species, and this represents the main reason why this work was begun. Also, given the record on the phytochemical analysis of the male cones of this species, which has evidenced some important differences between two years [3,5], the phytochemical analysis on the ethanolic extract of the leaves was repeated. The same extract was then analyzed for its potential radical scavenging, chelating, reducing antiglycative, antimicrobial, and antifungal activities as well as for the inhibitory effects on the production of aflatoxin B1 in *Aspergillus flavus* Link. In this paper, all these results were presented and discussed in relation to phytochemical content.

## 2. Results and Discussion

### 2.1. Phytochemical Analysis

**Figure 1:**
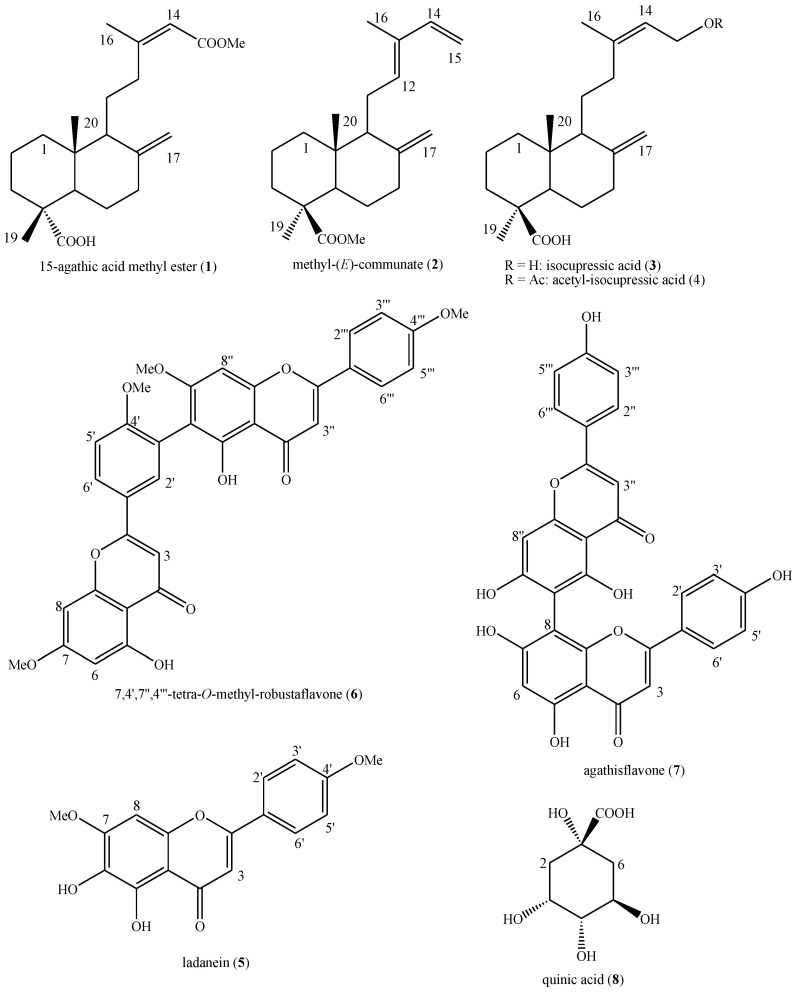
Structures of the identified compounds in the leaves of *Wollemia nobilis* W.G.Jones, K.D.Hill & J.M.Allen collected in the Botanical Garden of Rome.

The new phytochemical analysis of the leaves of *W. nobilis* collected in the Botanical Garden of Rome led to the identification of eight known secondary metabolites, namely, 15-agathic acid methyl ester (**1**), methyl-(*E*)-communate (**2**), isocupressic acid (**3**), acetyl-isocupressic acid (**4**), ladanein (**5**), 7,4′,7″,4‴-tetra-*O*-methyl-robustaflavone (**6**), agathisflavone (**7**) and quinic acid (**8**) (Figure 1).

These compounds belong to four different major classes of natural compounds, i.e., diterpenoids (**1**–**4**), flavonoids (**5**), biflavonoids (**6**–**7**) and organic acids (**8**).

To the best of our knowledge, 15-agathic acid methyl ester (**1**) and ladanein (**5**) were identified in the species for the first time during this study. In addition, methyl-(*E*)-communate (**2**), 7,4′,7″,4‴-tetra-*O*-methyl-robustaflavone (**6**), agathisflavone (**7**) and quinic acid (**8**) were identified in the leaf tissue of the species for the first time during this study. In fact, compounds (**1**, **5**) have previously been reported only in other species of the Araucariaceae family [7,8], whereas compounds (**2**, **6**–**8**) have been previously identified in other organs of the species [2,3,4,5,6] as well as in other species of the family [8]. Indeed, the other compounds (**3**–**4**) have been reported in the species and in this organ in past phytochemical studies [1,2,3,4,5,6] as well as in other species of the family [8]. Their presence is extremely important since it confirms their normal biosynthesis from this species and clearly displays the taxonomic correlations within the Araucariaceae family given that common compounds have been identified.

At the spectrophotometric analysis, the extract was found to be notably rich in tannins (expressed as tannic acid equivalents), which accounted for approximately two-thirds of the total polyphenol content (Table 1). Moreover, total flavonoids (expressed as quercetin equivalents) achieved about a 21.0% *w*/*w* of the extract (Table 1).

### 2.2. Antioxidant and Antiglycative Properties

**Figure 2:**
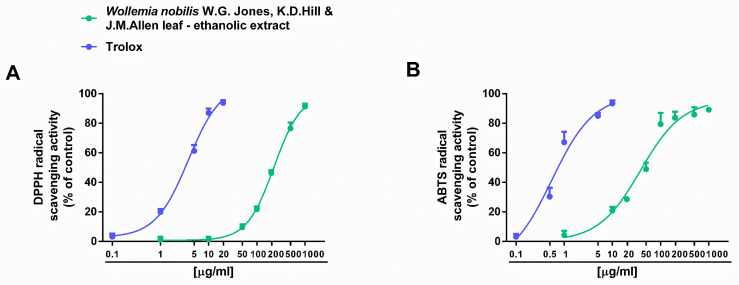
Scavenger activity of the ethanolic extract obtained from the leaf extract from *Wollemia nobilis* W.G.Jones, K.D.Hill & J.M.Allen and the positive control Trolox against DPPH^·^ (**A**) and ABTS^·+^ (**B**) radicals (n = 6).

The ethanolic extract from the leaves of *W. nobilis* was assessed for both direct and indirect antioxidant properties as well as for the antiglycative power. To this end, the scavenging effects towards synthetic DPPH^·^ and ABTS^·+^ radicals, iron chelating and reducing activities, and the inhibition of AGE generation were evaluated. The extract was able to neutralize both DPPH^·^ and ABTS^·+^ radicals (Figure 2), although with almost a six-fold higher potency against ABTS^·+^, as confirmed by IC_50_ values (Table 2). Indeed, complete radical neutralization was achieved at 1000 μg/mL for DPPH^·^ radicals (Figure 2A) and at 200 μg/mL for ABTS^·+^ (Figure 2B).

Considering the diverse reactivity of DPPH^·^ and ABTS^·+^ radicals, with DPPH^·^ being primarily neutralized by small molecules and ABTS^·+^ by both lipophilic and hydrophilic hydrogen atom donors, our hypothesis is that the higher potency of the extract against ABTS^·+^ could arise from the presence of a diverse pool of hydrophilic and lipophilic compounds with varying molecular sizes. Among them, radical scavenging properties against both radicals have been reported for agathisflavone (**7**) [9] and quinic acid (**8**) [10]. Similarly, ladanein (**5**) has been found to scavenge DPPH^·^, although at high concentrations [11]. We hypothesize the involvement of these compounds in the radical scavenging properties of the extract. However, the contribution of other primary compounds is also expected.

**Figure 3:**
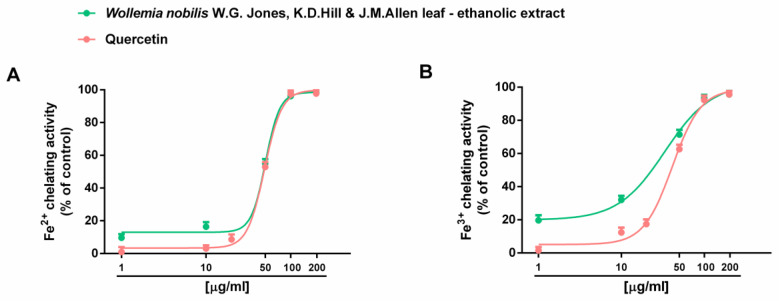
Chelating activity of the ethanolic extract obtained from the leaves of *Wollemia nobilis* W.G.Jones, K.D.Hill & J.M.Allen and of the positive control quercetin against ferrous (**A**) and ferric (**B**) ions (n = 6).

Under our experimental conditions, *W. nobilis* ethanolic leaf extract also exhibited marked chelating abilities towards both ferrous and ferric ions (Figure 3); conversely, the reducing power was almost null (Figure S1).

The comparison of IC_50_ values showed that the chelating activity of the extract was like that of the positive control quercetin, with a slightly higher potency against ferric ions than ferrous ones (Table 2).

**Figure 4:**
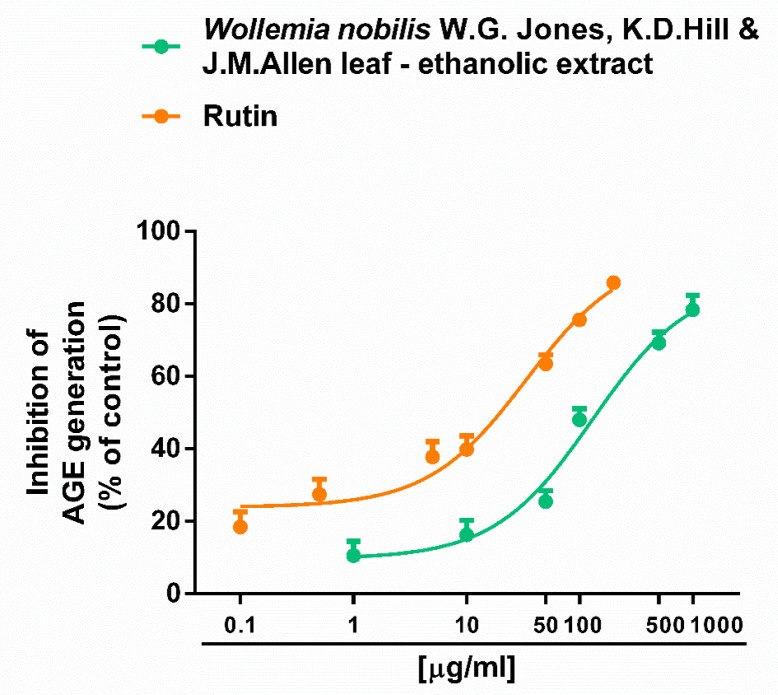
Antiglycative activity of the ethanolic extract obtained from the leaves of *Wollemia nobilis* W.G.Jones, K.D.Hill & J.M.Allen and of the positive control (n = 6).

Moreover, the extract completely inhibited the generation of AGE species (Figure 4), although with almost a five-fold lower potency than the positive control rutin.

This ability appeared at a three-fold higher concentration than the radical scavenging and chelating ones (Table 2).

Among the identified compounds, ferric ion chelating properties have already been reported for ladanein (**5**), related to the presence of a β-hydroxy-ketone group in its structure [12]. Moreover, diverse phenolic compounds have been studied for their metal chelating ability [13]. Also in this case, future studies will have to individuate other possible contributing compounds.

The Pearson correlation analysis showed that the scavenging activity of both DPPH^·^ and ABTS^·+^ radicals and ferrous and ferric ion chelation were significantly (at least *p* < 0.05) correlated with each other, as well as with the inhibition of AGE generation, except for ferric chelation with DPPH^·^ radical scavenger activity and with AGE generation (Table 3).

Altogether, this evidence suggests that many compounds, including flavonoids, bioflavonoids, organic acids and diterpenes, may contribute to the bioactivities of the extract. Among them, agathisflavone (**7**), which has been reported to possess neuroprotective properties through antioxidant and anti-inflammatory mechanisms [14,15,16,17], and the flavone ladanein (**5**), which exhibits antioxidant, antiproliferative and antiviral properties [12,18,19], suggest a future interest in *W. nobilis* extracts in cytoprotective strategies. Nonetheless, further specific investigations are required to elucidate the true pharmacological impact of these extracts and the precise role of their identified phytochemicals.

### 2.3. Antimicrobial Effect

**Figure 5:**
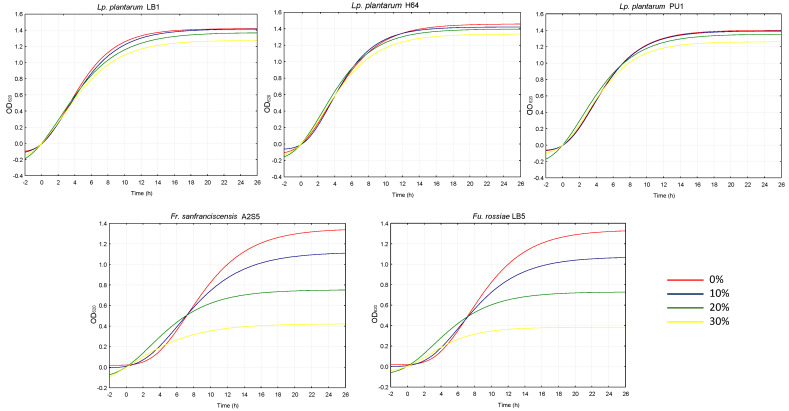
Growth kinetics of lactic acid bacteria cultivated in MRS broth supplemented with 0, 10, 20 and 30% of *W. nobilis* leaf ethanolic extract.

Aiming at investigating the potential antimicrobial activity of *W. nobilis* leaves, their ethanol extract was mixed at three different percentages with growth media to evaluate inhibition against several bacteria, yeasts and fungi used as a starter in the food industry or considered either spoilage or pathogenic microorganisms. As shown in Figure 5, each strain behaved differently in the presence of the extract. More specifically, among lactic acid bacteria, the growth of *Lactiplantibacillus plantarum* strains appeared unimpaired by the extract, regardless of the percentage used. On the contrary, *Furfurilactobacillus rossiae* LB5 and *Fructilactobacillus sanfranciscensis* A2S5 growth was significantly (*p* < 0.05) impacted by the extract proportionally to its addition. Indeed, the latency phase (λ) and the velocity of growth (µ_max_) were, respectively, higher and lower when the two strains were cultivated in the presence of the extract, compared to the respective controls. It is possible that the phenolic compounds in the extract highly affect the growth of *Furfurilactobacillus rossiae* and *Fructilactobacillus sanfranciscensis* more than that of *Lactiplantibacillus plantarum* strains, which are known to possess several enzymes to metabolize phenolic compounds [19].

Similarly, the extract was tested on conventional and unconventional yeasts used for the fermentation of several types of food and beverages (e.g., bread, wine, beer). The growth of *Saccharomyces cerevisiae*, undeniably the most studied and widely used eukaryote in a broad variety of industrial processes [20], was not affected by the addition of *W. nobilis* extract, not even at the highest percentage used (30%).

**Figure 6:**
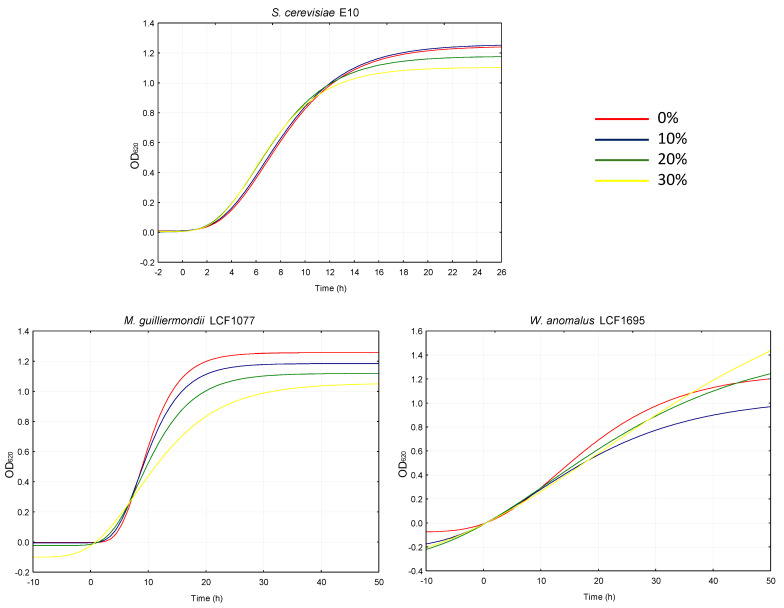
Growth kinetics of yeasts cultivated in their respective media supplemented with 0, 10, 20 and 30% of *W. nobilis* leaf ethanolic extract.

On the contrary, the µ_max_ recorded for *Meyerozyma guilliermondii* LCF1077 and *Wickerhamomyces anomalus* LCF1695 was inversely proportional to the supplementations (Figure 6). Nevertheless, the lambda decreased for both yeast species as the extract increased.

It is hypothesized that although the presence of phenolic compounds inhibits their growth, other substances (e.g., sugars) might have facilitated the adaptation stage. In this context, ladanein (**5**) has already demonstrated promising antimicrobial effects [12] as well as labdane diterpenoids [21], dimeric flavonoids [22] and quinic acid [23]. In fact, dimeric flavonoids are known to target bacterial and fungal cells by interacting with cell membranes and inhibiting their essential enzymes [22]. Anyway, there are limited studies for all the possible compounds and strains, and futures studies will surely have to focus on this aspect.

**Figure 7:**
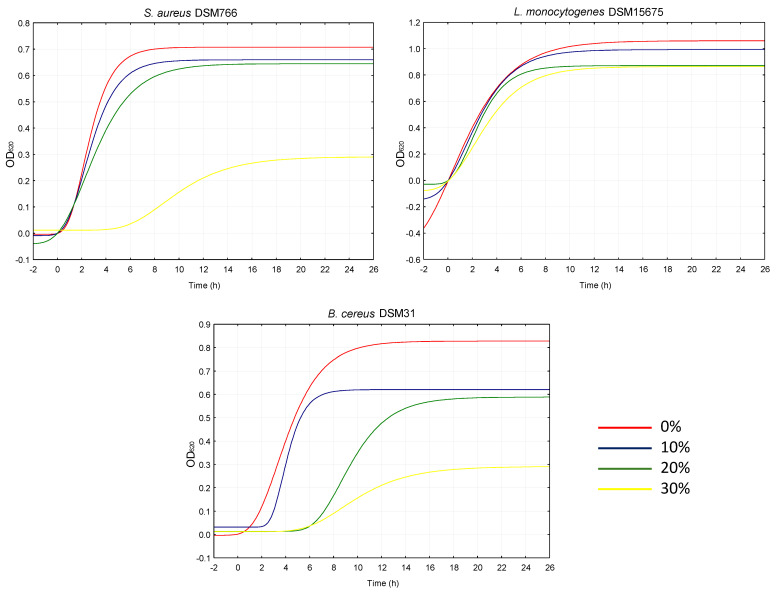
Growth kinetics of food pathogenic bacteria cultivated in their respective media supplemented with 0, 10, 20 and 30% of *W. nobilis* leaf ethanolic extract.

The control of foodborne pathogens through natural antimicrobial compounds that could extend food shelf-life is a current topic of research; hence, the extract was also tested against food pathogenic bacteria. *W. nobilis* inhibited the growth of all pathogens tested, especially *Bacillus cereus* DSM31 and *Staphylococcus aureus* DSM799 (Figure 7). Compared to the control (0%), decreases from 18 to 94% were observed for the cell density variation between the inoculum and stationary phase (A) when the extract was used at the maximum percentage.

It is possible that the compounds identified in the extract inhibited the growth of by damaging the bacterial cell membrane. Indeed, when the antibacterial activity of quinic acid against *Staphylococcus aureus* was investigated, it was found that quinic acid induces membrane hyperpolarization and decreases membrane fluidity, leading to compromised membrane integrity [24].

**Figure 8:**
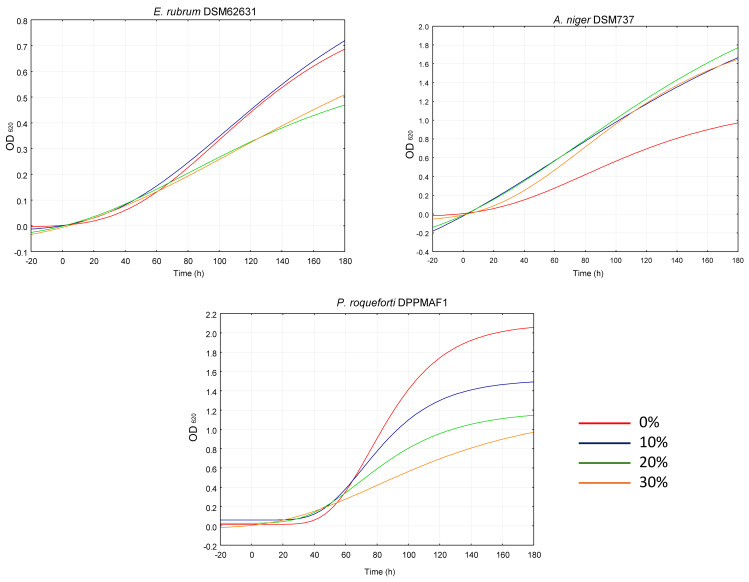
Growth kinetics of spoilage fungi cultivated in their respective media supplemented with 0, 10, 20 and 30% of *W. nobilis* leaf ethanolic extract.

As for the other classes of indicator microorganisms, fungi species behaved differently to the extract (Figure 8). *Penicillium roqueforti* DPPMAF1 was the most affected, showing up to 75 and 61% lower µ_max_ and A, respectively, compared to the control. *Eurotium rubrum* DSM62631 was inhibited only at additions of 20 and 30% whereas *Aspergillus niger* DSM737 growth was not affected by the extract.

Indeed, although plant phytochemicals, including phenolics, terpenoids, saponins, and alkaloids, are known for their antifungal activity [25], such effects are species dependent.

### 2.4. Antifungal and Inhibitory Effects on the Production of Aflatoxin B1 in Aspergillus flavus Link 3375

To assess the effects of *W. nobilis* leaf ethanolic extract on the phytopathogen *Aspergillus flavus* Link 3375, the extract was mixed at three different concentrations to evaluate the inhibition on *A. flavus* growth and AFLAB1 production.

**Figure 9:**
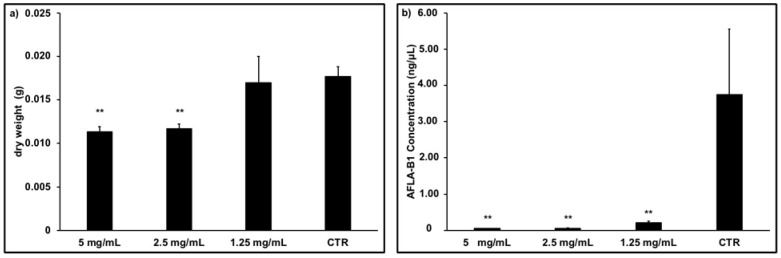
Dry weight of *A. flavus* mycelium (**a**) and AFLAB1 concentration (**b**) after 7 days in presence of *W. nobilis* leaf ethanolic extract at different concentrations (5, 2.5, 1.25 mg/mL). Statistical significance (** = *p* < 0.01) were examined by unpaired Student’s *t*-test.

As shown in Figure 9a, *A. flavus* growth was significantly impacted at two concentrations of *W. nobilis* extract (5 and 2.5 mg/mL), suggesting its antifungal capacity.

Investigations into the potential anti-mycotoxigenic activity also showed that *W. nobilis* extract plays a role in the biosynthesis of AFLAB1, and that, at all the extract concentrations tested, this was compromised (Figure 9b). The data represented are normalized on the mycelium weight.

The combinatory effect that interferes with the growth and AFLAB1 synthesis in *A. flavus* stimulates the understanding of the active principles that are able to act. As already reported, flavonoids may be involved in cell wall damage [26], while different terpenoids have inhibitory effects in the aflatoxin biosynthesis [27]. Therefore, the effects encountered are due to their presence [21,22] together with that of quinic acid (**8**) which has also demonstrated good antifungal effects [28]. Yet, also in this case, there are limited specific studies on all the compounds and future research will surely have to develop this knowledge.

## 3. Materials and Methods

### 3.1. Plant Material

Leaves of *W. nobilis* were collected in the Botanical Garden of Rome, located in Largo Cristina di Svezia, 23 A–24, 00165 Roma RM (geographical coordinates: 41°53′32 N, 12°27′57″ E), in July 2022. Botanical identification was performed by the botanist of the park and one of the authors (Dr. Claudio Scintu) by comparing the morphological features with those available in the literature [29]. A representative sample of this collection is stored in our laboratory for further reference under the voucher code WN18072022.

### 3.2. Chemicals

The following materials and solvents were used during this study: 96% ethanol for the extraction procedure; *n*-butanol, distilled water, dichloromethane and methanol as pure solvents or in a mixture of all of them as eluting systems for column chromatography separation on silica gel (40–63 μm), used as stationary phase; 2N sulfuric acid for the development of TLCs; deuterated solvents (CDCl_3_, CD_3_OD) for the identification of metabolites by means of NMR spectroscopy; Folin–Ciocâlteau phenol reagent, tannic acid, sodium carbonate, polyvinylpyrrolidone (PVP), aluminum chloride hexahydrate and quercetin for the quantification of polyphenols, tannins and flavonoids; 1,1-diphenyl-2-picryl-hydrazyl radical (DPPH^·^, 95% purity) and 2,2′-azo-(2-methylpropionamidine) dihydrochloride (AAPH; 98% purity) to assess the scavenging activity against DPPH^·^ and ABTS^·+^ radicals; iron(II) sulfate heptahydrate (FeSO_4_ × 7H_2_O), iron(III) chloride (FeCl_3_ × 6H_2_O) and hydroxylamine hydrochloride to evaluate iron chelating and reducing activity; bovine serum albumin, glucose, fructose and sodium azide for the inhibition of AGE generation; trolox, rutin and quercetin as positive controls in the bioactivity assays.

All the solvents were of RPE purity grade, if not differently specified and together with the deuterated solvents, the TLCs and HPLC-grade methanol were purchased from Merck (St. Louis, MO, USA), whereas silica gel was purchased from Fluka Analytical (Bergamo, Italy). The substances for the antioxidant activity assays were all purchased from Merck (Milan, Italy).

### 3.3. Instrumentations

NMR spectra were recorded at 298 K on a Jeol JNM-ECZ 600R spectrometer (Jeol Ltd, Tokyo, Japan) with a magnet operating at 14.09 T, corresponding to a proton resonance frequency of 600.19 MHz and equipped with a Jeol multinuclear z-gradient inverse probe head. ^1^H NMR spectra were acquired with 32 transients, a spectral width of 9013.7 Hz (corresponding to 15 ppm) and 64 K data points for an acquisition time of 7.3 s. The recycle delay was set to 7.7 s to achieve complete resonance relaxation between successive scans. The chemical shifts were referenced to TMS (s, 0 ppm) for spectra in CDCl_3_ or to the internal solvent signal of CD_2_HOD (quintet, 3.31 ppm) for spectra in CD_3_OD.

### 3.4. Extraction, Separation and Identification of the Metabolites

250 g of leaves were extracted with 96% ethanol (about 800 mL) three times after a maceration of 7 days, each. Ethanol was evaporated at a reduced pressure at 50 °C. Throughout the concentration procedure, the pH was checked on litmus paper to verify that it was not too acid or basic (below 5.5 and above 8.5) because an extreme acidity or alkalinity might cause unwanted secondary reactions in the extract such as the hydrolysis of ester and glycosidic bonds. In this case, the pH was about 8. The obtained dried, dark green extract weighed 5.1 g.

An aliquot of this extract (2.3 g) was subjected to a first chromatographic separation on silica gel (92 g, ratio about 1:40 *w*/*w*). The eluting system consisted of a mixture of *n*-butanol and distilled water at the concentration ratio of 82:18 *v/v* (400 mL). During the chromatographic run, the polarity of the eluting system was raised to allow the elution of the most polar compounds, using a mixture of *n*-butanol, methanol and distilled water at the concentration ratio of 70:10:30 *v/v/v* (300 mL). From this chromatographic separation, no compound could be clearly identified, and, for this reason, a second chromatographic step was conducted from the assembly of fractions 2–33 with a total weight of 1.131 g. The initial eluting system consisted of a mixture of dichloromethane and methanol at the concentration ratio of 98:2 *v/v* (100 mL), but, during the chromatographic run, the eluting system was modified in order to raise its polarity and allow the elution of the most polar compounds, using a mixture of dichloromethane and methanol at the concentration ratios of 95:5 *v/v* (200 mL), 9:1 *v/v* (150 mL), 8:2 *v/v* (200 mL), 7:3 *v/v* (150 mL) and 6:4 *v/v* (150 mL). From this chromatographic separation, all of the following compounds were identified: 15-agathic acid methyl ester (**1**) [30], methyl-(*E*)-communate (**2**), isocupressic acid (**3**) and acetyl-isocupressic acid (**4**) [3] in a mixture (ratio not calculable) from the assembly of fractions 1–62 with a total weight of 72.8 mg; ladanein (**5**) in a mixture with 7,4′,7″,4‴-tetra-*O*-methyl-robustaflavone (**6**) [7] (ratio 1:10 *w*/*w*) from the assembly of fractions 73–80 with a total weight of 28.8 mg; agathisflavone (**7**) [31] as an almost pure compound from the assembly of fractions 87–88 for the weight of 4.5 mg; quinic acid (**8**) [32] in mixture with saccharides and amino acids (ratio not calculable) from the methanol column wash with a total weight of 115.6 mg.

### 3.5. Spectroscopic Data of the Identified Compounds in W. nobilis Leaf Ethanol Extract

15-agathic acid methyl ester (**1**): ^1^H NMR (CDCl_3_, 600 MHz) δ: 5.64 (1H, d, *J* = 1.2 Hz, H-14), 4.87 (1H, br. s, H-17a), 4.52 (1H, br. s, H-17b), 3.68 (3H, s, COOMe), 2.15 (3H, s, Me-16), 1.12 (3H, s, Me-19), 0.73 (3H, s, Me-20).

Methyl-(*E*)-communate (**2**): ^1^H NMR (CDCl_3_, 600 MHz) δ: 6.34 (1H, dd, *J* = 18.2/11.3, H-14), 5.34–5.30 (1H, overlapped, H-12), 5.26–5.22 (1H, overlapped, Ha-15), 5.08 (1H, br. s, Ha-17), 4.94–4.90 (1H, overlapped, Hb-15), 4.50 (1H, br. s, Hb-17), 3.48 (3H, s, COOMe), 1.69 (3H, s, Me-16), 1.23 (3H, s, Me-19), 0.65 (3H, s, Me-20).

Isocupressic acid (**3**): ^1^H NMR (CDCl_3_, 600 MHz) δ: 5.39–5.35 (1H, overlapped, H-14), 4.86 (br. s, Ha–17), 4.51 (br. s, Hb–17), 4.15 (2H, d, *J* = 6.5, H-15), 1.68 (3H, s, Me-16), 1.25 (3H, s, Me-19), 0.60 (3H, s, Me-20).

Acetyl-isocupressic acid (**4**): ^1^H NMR (CDCl_3_, 600 MHz) δ: 5.34–5.30 (1H, overlapped, H-14), 4.87 (1H, br. s, Ha-17), 4.57 (2H, d, *J* = 7.1, H-15), 4.51 (1H, br. s, Hb–17), 2.08 (3H, s, Ac), 1.69 (3H, s, Me-16), 1.26 (3H, s, Me-19), 0.61 (3H, s, Me-20).

Ladanein (**5**): ^1^H NMR (CDCl_3_, 600 MHz) δ: 7.41 (1H, d, *J* = 9.1 Hz, H-2′ and H-6′), 6.85 (2H, d, *J* = 9.1 Hz, H-3′ and H-5′), 6.61 (1H, s, H-3), 6.57 (1H, s, H-8), 3.80 (3H, s, 4′-OMe), 3.78 (3H, s, 7-OMe).

7,4′,7″,4‴-tetra-*O*-methyl-robustaflavone (**6**): ^1^H NMR (CDCl_3_, 600 MHz) δ: 7.97 (1H, dd, *J* = 8.9/2.4 Hz, H-6′), 7.87 (1H, d, *J* = 2.4 Hz, H-2′), 7.46 (2H, d, *J* = 8.9 Hz, H-2‴ and H-6‴), 7.16 (1H, d, *J* = 8.9 Hz, H-5′), 6.82 (2H, d, *J* = 8.9 Hz, H-3‴and H-5‴), 6.65 (1H, s, H-3″), 6.61 (1H, s, H-3), 6.52 (1H, s, H-8″), 6.42 (1H, d, *J* = 2.3 Hz, H-8), 6.37 (1H, br. s, H-6), 3.85 (3H, s, 4‴-OMe), 3.82 (3H, s, 7-OMe), 3.79 (3H, s, 4′-OMe), 3.77 (3H, s, 7″-OMe).

Agathisflavone (**7**): ^1^H NMR (CD_3_OD, 600 MHz) δ: 7.98 (2H, d, *J* = 8.5 Hz, H-2′ and H-6′), 7.50 (2H, d, *J* = 8.3 Hz, H-2‴ and H-6‴), 6.98 (2H, d, *J* = 8.5 Hz, H-3′ and H-5′), 6.97 (2H, *J* = 8.3 Hz, H-3‴ and H-5‴), 6.76 (1H, s, H-8), 6.74 (1H, s, H-3), 6.61 (1H, s, H-3’’), 6.36 (1H, s, H-6’’).

Quinic acid (**8**): ^1^H NMR (CD3OD, 600 MHz) δ: 4.05–4.00 (1H, m, H-4), 3.98–3.94 (1H, m, H-5), 3.62–3.57 (1H, m, H-3), 2.10–1.98 (4H, m, Ha-2, Hb-2, Ha-6, Hb-6).

### 3.6. Spectrophotometric Determination of Total Polyphenols, Tannins and Flavonoids

The total content of polyphenols and tannins was measured by the Folin–Ciocâlteau method, as previously described [33]. Specifically, 20 μL of the tested extract and 100 μL of Folin–Ciocâlteau reagent were mixed, and then 80 μL of a sodium carbonate solution (7.5% *w/v*) were added and shaken. After a 2 h incubation, the absorbance at 765 nm was determined using a microplate reader (Epoch Microplate Spectrophotometer, BioTek, AHSI, Milan, Italy). Tannins were precipitated by adding 100 mg of polyvinylpyrrolidone per mL of extract; thereafter, the amount was calculated as the difference between the polyphenol content in the extract and that in the supernatant. The total content of polyphenols and tannins was expressed as tannic acid equivalents (TAEs) per mg of extract, using the tannic acid calibration curve (Y = 42,420X + 0.0048; r^2^ = 0.998).

To assess the total flavonoid content, 100 μg/mL of the extract and aluminum trichloride solution (2% *w/v* in methanol) were combined and incubated for 10 min, then the absorbance was measured at 415 nm. The total flavonoid content was obtained from the calibration curve of quercetin (Y = 13,741X + 0.01541; r^2^ = 0.998) and expressed as quercetin equivalents (QEs).

### 3.7. Antioxidant Assays

#### 3.7.1. Radical Scavenging Activity

To evaluate the scavenging activity of the tested extract against DPPH^·^ and ABTS^·+^ radicals, previously established methods with slight modifications were applied [34].

For the DPPH^·^ radical scavenger activity assay, a 20 µL serial dilution of the sample was added to 180 µL of a 0.1 mM DPPH^·^ radical solution, and then the mixture was incubated in the dark at room temperature for 30 min.

To assess radical scavenging power against ABTS^·+^ radicals, a preliminary activation, combining equal volumes of 5 mM ABTS^·+^ and 2 mM AAPH solutions in phosphate-buffered saline (PBS) and incubating the mixture at 68 °C for 45 min, was performed. Subsequently, 20 µL serial dilutions of the extract and 180 µL of the ABTS^·+^ radical cation were mixed and incubated in the dark at 37 °C for 10 min.

Absorbances of DPPH^·^ and ABTS^·+^ radicals were read at 517 nm and 734 nm, respectively, using an Epoch microplate spectrophotometer (BioTek, AHSI, Milan, Italy).

Suitable controls were included in each assay to determine the maximum radical absorbance (negative control) and to detect potential interfering compounds in the samples, with trolox serving as a positive control. The scavenging activity was expressed as a percentage relative to control.

#### 3.7.2. Iron Chelating Activity

The chelating abilities of the extract were assessed towards both ferrous and ferric ions, using the ferrozine assay, as previously reported [34].

Treatments with vehicle, corresponding to a lack of chelating activity, and quercetin were included as negative and positive controls, respectively. Additional controls were tested to evaluate potential interfering compounds in the samples.

To evaluate the ferrous chelating activity, equal volumes of a freshly prepared solution of FeSO_4_ × 7H_2_O (200 µM) and the extract were mixed for 2 min, and then 50 µL of acetate buffer and of a ferrozine solution (5 mM) were further added. Similarly, to determine ferric ion chelation, after mixing a solution of FeCl_3_ × 6H_2_O (200 µM) and the extract for 2 min, 50 µL of a hydroxylamine solution (5 mM) and of ferrozine solution (5 mM) were added. Thereafter, the absorbance was measured at 562 nm using an Epoch microplate spectrophotometer (BioTek, AHSI, Milan, Italy). The percentage of chelating activity was determined with respect to the vehicle.

#### 3.7.3. Ferric Reducing Activity

The ferrozine assay was applied to assess the ferric reducing power of the extracts, as previously reported [34].

To this end, a freshly prepared solution of FeCl_3_ × 6H_2_O (200 µM) and the extract were mixed for 2 min, and then 50 µL of acetate buffer and of a ferrozine solution (5 mM) were further added. Thereafter, the absorbance was measured at 562 nm using an Epoch microplate spectrophotometer (BioTek, AHSI, Milan, Italy). Suitable controls, including vehicle (corresponding to a lack of reducing activity), trolox (used to determine the maximum reducing effects) and additional treatments to detect potential interfering compounds in the samples, were assayed. The percentage of reducing activity was expressed with respect to trolox.

#### 3.7.4. Antiglycative Activity

The antiglycative power of the extract was evaluated in terms of inhibition of advanced glycation end-product (AGE) generation, using the standard phenolic rutin as a positive control, as previously reported [33].

To this end, progressive dilutions of the sample and a reaction mixture containing bovine serum albumin (150 mM), phosphate buffer (50 mM; pH 7.4), sodium azide (0.02% *w*/*v*), fructose (0.4 M) and glucose (0.4 M) were mixed and incubated at 37 °C for 7 days. After incubation, the AGE fluorescence was measured at an excitation wavelength of 355 nm and an emission wavelength of 460 nm, using a Cytation 1 Cell Imaging Multimode Reader (BioTek, AHSI, Milan, Italy). The inhibitory activity was calculated as a percentage of the control.

#### 3.7.5. Statistical Analysis

Data were displayed as means ± SEs of at least three experiments, each one with at least three replicates. Statistical analysis was performed using GraphPad Prism™ (Version 4.00) software (GraphPad Software, Inc., San Diego, CA, USA). Differences between treatments were determined with one-way analysis of variance (one-way ANOVA), followed by Dunnett’s multiple comparison post test; a *p* value < 0.05 was considered as significant. IC_50_ (half maximal inhibitory concentration) was determined from the concentration–response curves using the Hill equation, as previously reported [12].

### 3.8. Microorganisms and Cultivation

Bacteria, yeasts and fungi commonly used as starters for the food industry or considered contaminants or food pathogens were used to assess the antimicrobial activity of *W. nobilis*. Lactic acid bacteria strains (*Lactiplantibacillus plantarum* H64, *L. plantarum* PU1, *L. plantarum* LB1, *Fructilactobacillus sanfranciscensis* A2S5 and *Furfurilactobacillus rossiae* LB5) were routinely propagated on De Man Rogosa and Sharpe (MRS, Oxoid Ltd., Basingstoke, Hampshire, UK) at 30 °C.

*Listeria monocytogenes* DSM15675 was cultivated on *Luria–Bertani* (Oxoid, Basingstoke, UK) at 37 °C, whereas *Bacillus cereus* DSM31 and *Staphylococcus aureus* DSM799 were propagated on Tryptone Soya Yeast Extract (TSYE, Merck) at 30 and 37 °C, respectively.

Among yeasts, *Saccharomyces cerevisiae* E10 was cultivated on Sabouraud (Sab, Oxoid) at 30 °C, whereas *Meyerozyma guilliermondii* LCF1077 and *Wickerhamomyces anomalus* LCF1695 were propagated on Malt Extract (ME, Oxoid) agar at 25 °C.

*Penicillium roqueforti* DPPMAF1, *Aspergillus niger* DSM737 and *Eurotium rubrum* DSM62631 were routinely propagated on Potato Dextrose (PD, Oxoid) agar at 25 °C.

### 3.9. Antimicrobial Screening

To assess the antimicrobial activity of the extract, the micro-titer plate assay, as previously proposed by Nionelli et al. [35], was used.

A total of 3 g of lyophilized *W. nobilis* leaf extract was resuspended in 60 mL of distilled water, dissolved with a magnetic stirrer and sterilized using 0.2 μm sterile syringe filters. Then, indicator microorganisms were cultivated as described above, using the respective liquid media supplemented with 0, 10, 20, and 30% of resuspended extract using 96-multiwell plates. Each well contained 200 μL of medium and the inoculum, which was at 4% for bacteria and yeasts and 10 μL of a 105 condia/mL for fungi. Before the analysis, the pH of the media was checked to ensure the pH of the extract added could not interfere with the growth of the strains. Multiwell plates were incubated at 25, 30 or 37 °C depending on the indicator tested, and growth was monitored for 24, 48 and 72 h using a spectrophotometer (Microplate reader, AMR-100, Hangzhou Allsheng Instruments Co., Ltd., Hangzhou, China) set at 620 nm.

The analysis was carried out in triplicate and data were subjected to one-way ANOVA; pair comparison of treatment means was obtained by Tukey’s procedure at *p* < 0.05, using statistical software Statistica 12.5 (StatSoft Inc., Tulsa, OK, USA). Growth kinetics were modeled by a non-linear regression procedure according to the Gompertz equation as modified by Zwietering et al. [36]: y = k +A exp {−exp[(µ_max_ e/A)(λ − t) + 1]}, where y is the OD620; k is the initial level of the dependent variable to be modeled (OD620); A is the cell density variation (between inoculation and the stationary phase); µ_max_ is the maximum growth rate expressed as OD620 units/h; and λ is the length of the lag phase measured in hours. The experimental data were modeled using Statistica 12.5 software.

### 3.10. Aspergillus flavus Growth and AFLAB1 Production and Analysis

The *Aspergillus flavus* Link 3375 strain was grown on a Potato Dextrose agar (Himedia, Maharashtra, India) plate at 28 °C. To set up the 96-multiwell test, a spore suspension was prepared at a concentration of 1 × 105 spores/mL using a Thoma chamber and observed with a light microscope (Zeiss Axio Scope, Carl Zeiss, Göttingen, Germany). A volume of 10 μL of the spore suspension was inoculated in 190 µL of Potato Dextrose broth (Himedia, Maharashtra, India).

Nine replicates per sample were incubated with different concentrations (5, 2.5 and 1.25 mg/mL) of *W. nobilis* extract for 7 days. The extract was sterilized by filtration using a 0.45 μm filter. After the incubation period, the 96-mutiwell plate was lyophilized and the mycelium was weighed. For aflatoxin extraction, samples were mixed with 1000 µL of acetonitrile, water and acetic acid in a ratio of 70:29:1 *v*/*v*/*v* and subjected to 20 min of shaking using a TissueLyser (Qiagen, Hilden, Germany). After centrifugation at 1000 rpm for 10 min, the supernatant was carefully transferred to a new tube. The extracts were evaporated under an air stream and analyzed with HPLC-MS^n^ (Agilent 1200 Infinity coupled with Agilent 6400 Series Triple Quadrupole LC/MS System, Agilent Technologies, Inc., Santa Clara, CA, USA). Chromatographic separation and acquisition were achieved following the method reported in Frezza et al. [37].

## 4. Conclusions

Our study on the ethanolic extract from the leaves of *W. nobilis* collected in the Botanical Garden of Rome led to the identification of eight known secondary metabolites: 15-agathic acid methyl ester (**1**), methyl-(*E*)-communate (**2**), isocupressic acid (**3**), acetyl-isocupressic acid (**4**), ladanein (**5**), 7,4′,7″,4‴-tetra-*O*-methyl-robustaflavone (**6**), agathisflavone (**7**) and quinic acid (**8**). To the best of our knowledge, compounds (**1**) and (**5**) were identified here for the first time in this species, while compounds (**2**) and **(6**–**8**) are reported for the first time in its leaf tissue. The extract also exhibited promising radical scavenging, chelating, antiglycative, antimicrobial and antifungal activities, as well as a notable inhibitory effects on the production of aflatoxin B1 by *A. flavus*. Although the identified metabolites may contribute to the observed effects, we cannot exclude that the synergistic interactions within the phytocomplex may be responsible for the biological activities of the extract. Altogether these findings reinforce the taxonomic correlation within the Araucariaceae family and underline the need to perform further phytochemical and pharmacological studies on *W. nobilis* to better characterize its potential pharmaceutical value.

## Figures and Tables

**Table 1:** Amounts of total polyphenols and tannins, expressed as tannic acid equivalents (TAE), and flavonoids, expressed as quercetin equivalents (QE), in the ethanolic extract from the leaves of *Wollemia nobilis* W.G.Jones, K.D.Hill & J.M.Allen (n = 6).

Classes of Compounds	µg/mg Extract (Mean ± SE)
Polyphenols (TAE)	48.89 ± 5.51
Tannins (TAE)	33.72 ± 5.80
Flavonoids (QE)	210.10 ± 16.31

**Table 2:** IC_50_ values of the ethanolic extract obtained from the leaves of *Wollemia nobilis* W.G.Jones, K.D.Hill & J.M.Allen and the positive controls in the antioxidant activity assays.

Antioxidant Assay	*Wollemia nobilis* Leaf Extract	Positive Controls
	IC_50_ (CL) μg/mL	
DPPH^·^ radical scavenger activity	227.0 (180.8–284.9)	3.8 (2.7–5.3) ^a^
ABTS^·+^ radical scavenger activity	39.7 (27.4–57.4)	0.6 (0.3–1.0) ^a^
Inhibition of AGE generation	142.4 (82.3–235.3)	33.7 (18.3–2.2) ^b^
Ferrous ion chelating activity	50.3 (47.3–53.6)	49.1 (46.2–52.1) ^b^
Ferric ion chelating activity	33.4 (25.6–43.7)	41.1 (36.3–43.4) ^b^
Ferric ion reducing activity	nd	7.0 (2.6–19.1) ^a^

CL: confidence limits. ^a^ Trolox. ^b^ Quercetin. nd, not determined due to negligible activity observed.

**Table 3:** Pearson correlation coefficient among antioxidant activity assays for the leaf extract from *Wollemia nobilis* W.G.Jones, K.D.Hill & J.M.Allen.

	Pearson r (CL; R Square)				
	DPPH^·^ Scavenger Activity	ABTS^·+^ Scavenger Activity	Ferrous Ion Chelating Activity	Ferric Ion Chelating Activity	Inhibition of AGE Generation
DPPH**^·^** scavenger activity	-	0.81 * (0.15–0.99; 0.66)	0.87 (0.31–0.99; 0.77)	0.87 (0.93–0.99; 0.76)	0.96 ** (0.68–0.99; 0.92)
ABTS**^·+^** scavenger activity	0.81 * (0.15–0.99; 0.66)	-	0.98 ** (0.71–0.99; 0.96)	0.99 ** (0.03–0.99; 0.98)	0.94 ** (0.54–0.99; 0.88)
Ferrous ion chelating activity	0.87 (0.31–0.99; 0.77)	0.99 *** (0.90–0.99; 0.99)	-	0.99 ** (0.81–0.99; 0.99)	0.98 * (0.42–0.99; 0.97)
Ferric ion chelating activity	0.87 (0.93–0.99; 0.76)	0.99 ** (0.03–0.99; 0.98)	0.99 *** (0.89–0.99; 0.99)	-	0.95 (0.17–0.99; 0.89)
Inhibition of AGE generation	0.96 ** (0.68–0.99; 0.92)	0.94 ** (0.54–0.99; 0.88)	0.98 * (0.42–0.99; 0.97)	0.95 (0.17–0.99; 0.89)	-

CL: Confidence limits. * *p* < 0.05, ** *p* < 0.01 and *** *p* < 0.001, statistically significant correlation (two-tailed *t*-test).

## Data Availability

The original contributions presented in this study are included in the article. Further inquiries can be directed to the corresponding authors.

## Supplementary Materials

The following supporting information can be downloaded at: https://www.mdpi.com/article/10.3390/plants14081244/s1, Figure S1. Reducing activity of the ethanolic extract obtained from the leaves of *Wollemia nobilis* W.G.Jones, K.D.Hill & J.M.Allen and of the positive control trolox.
