# Supplementary material for: Phytochemical Analysis and Biological Activities of Wollemia nobilis W.G.Jones, K.D.Hill & J.M.Allen Leaves Collected in the Botanical Garden of Rome
Source: Plants (Basel). 2025 Apr 19;14(8):1244. doi: 10.3390/plants14081244 (PMC12030752; doi:10.3390/plants14081244)
Supplement: Supplementary file 1 [file plants-14-01244-s001.zip › plants-3568080-supplementary.pdf]

*plants*

MDPI

*Article*

# Phytochemical Analysis and Biological Activities of *Wollemia nobilis* W.G.Jones, K.D.Hill & J.M.Allen Leaves Collected in the Botanical Garden of Rome

**Claudio Frezza [1,*], Daniela De Vita [2], Ottavia Giampaoli [2], Marzia Beccaccioli [2], Michela Verni [2], Federica Violetta Conti [2], Laura Fonti [2], Marco Franceschin [3], Fabio Sciubba [2,4], Claudio Scintu [2], Letizia Corsetti [5], Antonella Di Sotto [5], Carlo Giuseppe Rizzello [2], Massimo Reverberi [2] and Fabio Attorre [2]**

[1] Dipartimento di Scienze della Vita, della Salute e delle Professioni Sanitarie, Università degli Studi Link Campus, Via del Casale di San Pio V, 44, 00165 Rome, Italy
[2] Dipartimento di Biologia Ambientale, Università di Roma "La Sapienza", 00185 Rome, Italy; daniela.devita@uniroma1.it (D.D.V.); ottavia.giampaoli@uniroma1.it (O.G.); marzia.beccaccioli@uniroma1.it (M.B.); michela.verni@uniroma1.it (M.V.); violetta.conti@uniroma1.it (F.V.C.); laurifonti49@gmail.com (L.F.); fabio.sciubba@uniroma1.it (F.S.); claudio.scintu@uniroma1.it (C.S.); carlogiuseppe.rizzello@uniroma1.it (C.G.R.); massimo.reverberi@uniroma1.it (M.R.); fabio.attorre@uniroma1.it (F.A.)
[3] Dipartimento di Chimica, Università di Roma "La Sapienza", 00185 Rome, Italy; marco.franceschin@uniroma1.it
[4] NMR-Based Metabolomics Laboratory (NMLab), Università di Roma "La Sapienza", Piazzale Aldo Moro 5, 00185 Rome, Italy
[5] Dipartimento di Fisiologia e Farmacologia "V. Erspamer", Università di Roma "La Sapienza", Piazzale Aldo Moro 5, 00185 Rome, Italy; letizia.corsetti@uniroma1.it (L.C.); antonella.disotto@uniroma1.it (A.D.S.)
* Correspondence: c.frezza@unilink.it

Academic Editor: Hazem Salaheldin Elshafie

Received: 20 March 2025
Revised: 5 April 2025
Accepted: 14 April 2025
Published: date

**Citation:** Frezza, C.; De Vita, D.; Giampaoli, O.; Beccaccioli, M.; Verni, M.; Conti, F.V.; Fonti, L.; Franceschin, M.; Sciubba, F.; Scintu, C.; et al. Phytochemical Analysis and Biological Activities of *Wollemia nobilis* W.G.Jones, K.D.Hill & J.M.Allen Leaves Collected in the Botanical Garden of Rome. *Plants* **2025**, *14*, x. https://doi.org/10.3390/xxxxx

**Copyright:** © 2025 by the authors. Submitted for possible open access publication under the terms and conditions of the Creative Commons Attribution (CC BY) license (https://creativecommons.org/licenses/by/4.0/).

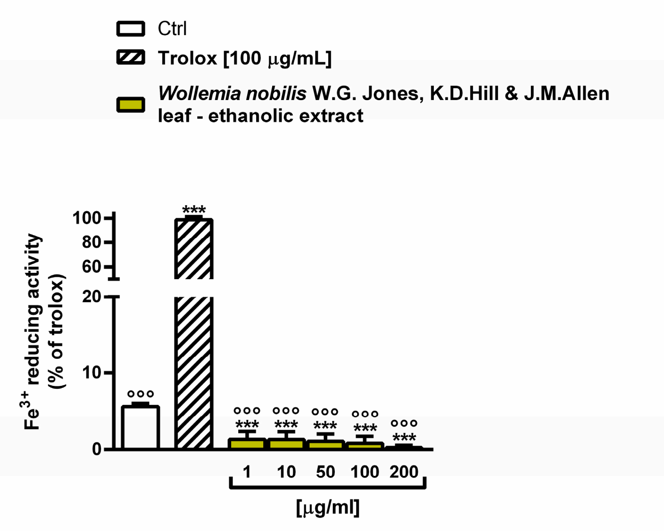


**Figure S1.** Reducing activity of the ethanolic extract obtained from the leaves of *Wollemia nobilis* W.G.Jones, K.D.Hill & J.M.Allen and of the positive control trolox. *** $p < 0.001$, statistically significant difference with respect to Ctrl (one-way ANOVA followed by Dunnett's multiple comparison post test). °°° $p < 0.001$, statistically significant difference with respect to Trolox (one-way ANOVA followed by Dunnett's multiple comparison post test).
